# Elaboration of Design and Optimization Methods for a Newly Developed CFRP Sandwich-like Structure Validated by Experimental Measurements and Finite Element Analysis

**DOI:** 10.3390/polym13244348

**Published:** 2021-12-12

**Authors:** György Kovács

**Affiliations:** Faculty of Mechanical Engineering and Informatics, Institute of Manufacturing Science, University of Miskolc, Egyetemváros, H-3515 Miskolc, Hungary; altkovac@uni-miskolc.hu

**Keywords:** CFRP laminate, new sandwich-like structure, experimental measurements, finite element method, mass and cost optimization method

## Abstract

Nowadays, the application of composite materials and light-weight structures is required in those industrial applications where the primary design aims are weight saving, high stiffness, corrosion resistance and vibration damping. The first goal of the study was to construct a new light-weight structure that utilizes the advantageous characteristics of Carbon Fiber Reinforced Plastic (CFRP) and Aluminum (Al) materials; furthermore, the properties of sandwich structures and cellular plates. Thus, the newly constructed structure has CFRP face sheets and Al stiffeners, which was manufactured in order to take experimental measurements. The second aim of the research was the elaboration of calculation methods for the middle deflection of the investigated sandwich-like structure and the stresses that occurred in the structural elements. The calculation methods were elaborated; furthermore, validated by experimental measurements and Finite Element analysis. The third main goal was the elaboration of a mass and cost optimization method for the investigated structure applying the Flexible Tolerance optimization method. During the optimization, seven design constraints were considered: total deflection; buckling of face sheets; web buckling in stiffeners; stress in face sheets; stress in stiffeners; eigenfrequency of the structure and constraints for the design variables. The main added values of the research are the elaboration of the calculation methods relating to the middle deflection and the occurred stresses; furthermore, elaboration of the optimization method. The primary aim of the optimization was the construction of the most light-weighted structure because the new light-weight sandwich-like structure can be utilized in many industrial applications, e.g., elements of vehicles (ship floors, airplane base-plate); transport containers; building constructions (building floors, bridge decks).

## 1. Introduction

Increasing market competition, fast changing customers’ demands and the pandemic situation results that companies have to put emphasis on the application of advanced materials, innovative structures and modern manufacturing technologies in order to maintain their competitiveness.

● FRP composites have many advantageous properties compared to metal materials, which are the following: low density, high strength, high vibration damping, chemical and corrosion resistance, high bending stiffness, good thermal insulation, advantageous design versatility, etc. [1,2].

One of the most important characteristics of the FRP materials is their low density, which causes significant weight saving compared to traditional materials, such as steel. Therefore, the FRP composites are widely used in those industrial applications where the primary design aim is weight saving, e.g., structural elements of transport vehicles; transport containers; chemical vessels; building constructions (building floors, elements of bridges or warehouses, etc.) [3,4,5].

The fiber reinforced composite materials consist of two different components, which are the fibers and the basic matrix. The fibers ensure the high strength. The basic matrix holds and protects the fibers from negative environmental impacts. There are many types of fibers (e.g., carbon, glass, aramid) and matrix (e.g., resins, ceramics) materials. The combination of these components is infinite, which results in high variety of composite materials for several special engineering applications [6,7,8,9,10].

The fiber reinforced laminated composite plates are a commonly used form of FRP composites. Fiber reinforced composite laminates are built up of more thin layers (laminae) stacked together. Laminated plates are also widely used in several industrial applications instead of metals due to their high strength and light weight [11,12].

In the literature, there are several publications that discuss the micro- and macromechanics of fiber reinforced laminated composite plates and their design methods. It can be concluded—based on the syntheses of the literature—that the calculation methods for the laminated composite materials and structures are more complex compared to homogenous materials [13,14].

There are a lot of articles that deal with the optimization of composite laminates and composite structures. The objective functions, design constraints and optimization algorithms are discussed in these papers [15,16,17]. The most often applied objective functions are the following: weight, deflection, eigenfrequency, sequence of layers in the laminate, etc. [18,19].

In the existing literature, there are several articles that discuss those methods which can be used for the validation of analytical calculations. These are the different experimental measurements and Finite Element simulations [20,21,22,23,24].

● The first goal of the study was to construct a new structure that utilizes the advantageous characteristics of Fiber Reinforced Plastic (FRP) and Aluminum (Al) materials; furthermore, the properties of sandwich structures and cellular plates.

A new construction was developed which has Aluminum square hollow section stiffeners and Carbon Fiber Reinforced Plastic (CFRP) face sheets (the investigated structure is introduced in Section 2). It can be concluded that the newly developed sandwich-like structure is the combination of different materials (CFRP and Al) and structural elements (elements of sandwich structures and cellular plates). Therefore, the integration of the advantages of different materials and structural elements is even more efficient than applying each of the materials and structural elements individually [25,26,27,28].

Both Aluminum and CFRP composite materials have low density, good corrosion resistance and high vibration damping. Thus, the combination of these advantageous characteristics results in more efficient solutions for special engineering applications. 

This structure is a sandwich-like structure because the face sheets made of FRP composite (as in the case of sandwich structures); furthermore, the stiffeners are made of metal (as in the case of cellular plates).

FRP composite sandwich structures generally build up from an inner core (which has low-density) and FRP face sheets. The most commonly used cores are foams or honeycomb structures. The most important characteristics of the FRP sandwich structures are the high strength to weight ratio, high vibration damping, high stiffness to weight ratio, good design versatility, easy and fast manufacturing [29,30,31,32]. There are several publications relating to the calculation methods, design, optimization and applications of FRP composite sandwich structures [33,34,35,36,37].

On the contrary, the cellular plates generally consist of metal face sheets and metal stiffeners welded into the face sheets. Due to the two structural elements (face sheets and stiffeners) the cellular plates have high strength; therefore, these structures are able to withstand higher loading conditions. Due to the complex geometry of the cellular structures, the design and optimization procedures of these structures are more complex compared to the design of monolithic structures [38,39,40].

● The phases of the research and the structure of the article are the following:

**(1)** The first goal of the study was to construct a new light-weight structure that utilizes the advantageous characteristics of Fiber Reinforced Plastic (FRP) and Aluminum (Al) materials; furthermore, the properties of sandwich structures and cellular plates.

A new construction was developed, which consists of Al square hollow section stiffeners and CFRP face sheets. The face sheets are riveted to the stiffeners. The newly constructed structure was manufactured in order to take experimental measurements (the developed new sandwich-like structure is introduced in Section 2).

**(2)** The second aim of the research was the elaboration of calculation methods on the one hand for the middle deflection of the new investigated sandwich-like structure; on the other hand, for the stresses that occurred in the structural elements. Before the elaboration of the calculation methods relating to the sandwich-like structure, preliminary calculations and experimental measurements had to be achieved relating to the different structural elements (face sheets, stiffeners and rivets). These preliminary calculations and measurements were discussed in Section 2.1, Section 2.2, Section 2.3.

After it, calculation methods were elaborated for the middle deflection of the investigated new sandwich-like structure and the stresses that occurred in the structural elements (Section 3.1). Then experimental measurements (Section 3.2) and Finite Element analysis (Section 3.3) were carried out. The comparison of the results of the calculations, measurements and Finite Element analysis showed good agreements which confirmed the correctness of the elaborated calculation methods (Section 3.4).

**(3)** The third main goal was the elaboration of an optimization method for the investigated newly developed light-weight sandwich-like structure.

In Section 4, the elaborated optimization method is introduced. The optimization was achieved by the Flexible Tolerance optimization method (Section 4.4). The elaborated mass and cost objective functions were applied during the optimization (Section 4.1 and Section 4.2). Furthermore, the following 7 design constraints were also considered (Section 4.3) during the optimization: (1.) total deflection; (2.) buckling of face sheet; (3.) web buckling in stiffeners; (4.) stress in the CFRP face sheets; (5.) stress in the stiffeners; (6.) eigenfrequency of the construction; (7.) size constraints relating to the design variables.

The cost objective function was elaborated based on the experiences gained during the manufacturing of the investigated structure. The elaborated calculation methods were applied during the formulation of the following design constraints: total deflection, stress in the face sheets and stress in the stiffeners.

● The main added value and novelty of the research are the following:

**(1)** Calculation methods were elaborated on the one hand for the middle deflection of the newly constructed sandwich-like structure; on the other hand, for the stresses that occurred in the structural elements (CFRP face sheets and Al stiffeners). Then experimental measurements and Finite Element analysis were carried out which validated the correctness of the elaborated calculation methods.

**(2)** The other novelty of my research is that a mass and cost optimization method was elaborated relating to the newly constructed sandwich-like structure. The optimization was achieved by the Flexible Tolerance optimization method considering seven design constraints. The primary aim of the optimization was to construct a minimal weight structure.

It can be concluded that there is a gap in the existing literature, because there cannot be found any publication which discusses either the above-mentioned elaborated new calculation methods (middle deflection; stresses occurred in the structural elements) or the elaborated optimization method for the newly constructed sandwich-like structure.

Therefore, the results of the study fill the gap in the recent literature which provides new theoretical information for the researchers. At the same time the newly constructed light-weight composite sandwich-like structure can be widely used in the practice (e.g., element of vehicles; transport containers; building constructions) which can be utilized by the end users.

## 2. Materials and Methods—Structural Components of the Analyzed Sandwich-like Structure

**Figure 1:**
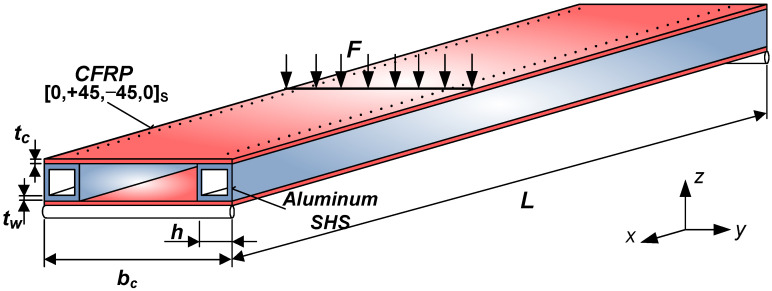
The investigated sandwich-like structure.

The newly constructed sandwich-like structure consists of CFRP face sheets and two Al square hollow section stiffeners. The face sheets are riveted to the stiffeners (Figure 1).

This structure is the combination of different materials (CFRP and Al) and structural elements (elements of sandwich structures and cellular plates). This construction is a sandwich-like structure, because the face sheets are made of FRP composite (as in the case of sandwich structures); furthermore, the stiffeners are made of metal (as in the case of cellular plates).

**Figure 2:**
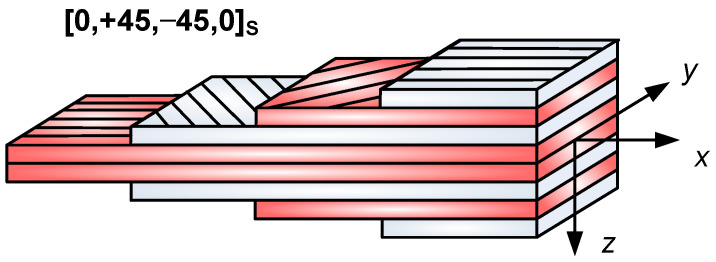
Layer sequence of the face sheet (8 layers).

The elaborated single-cellular sandwich-like structure is shown in Figure 1. The face sheets are manufactured from 8 laminae, with the following layer sequence [0, +45, −45, 0]_s_ (Figure 2). The face sheets are riveted to the Aluminum square hollow section (SHS) stiffeners (30 mm × 30 mm × 2 mm).

The investigated structure (*L* = 1200 mm, *b_c_* = 220 mm) is simply supported. The load (*F* = 500 N) acts on the middle line of the construction as a uniformly distributed line-load.

Due to the complexity of the investigated structure, the mechanical properties of the structure are difficult to define applying numerical and analytical approximations. Therefore, experimental measurements and Finite Element simulations were carried out to validate the calculation methods and their results.

### 2.1. CFRP Face Sheets as Structural Elements

The newly developed sandwich-like structure consists of 3 structural elements: (1.) CFRP face sheets; (2.) two Al square hollow section stiffeners and (3.) rivets for joining the CFRP face sheets and the Al stiffeners.

#### 2.1.1. CFRP Face Sheets—Calculated Elasticity Modulus and Flexural Modulus

The CFRP face sheets are manufactured from eight laminas with the following layer sequence [0, +45, −45, 0]_s_. The volume fraction of the carbon fiber is 55%, while the volume fraction of the Epoxy ES-67 matrix is 45% in a lamina. Of the carbon fibers, 84% are in longitudinal, while 16% of the fibers are in the transversal direction in a layer.

The material properties of the investigated laminate are the following: elasticity modulus of a CFRP layer in the longitudinal direction is *E_1_* = 54 GPa, elasticity modulus in transversal direction is *E_2_* = 40 GPa and the shear modulus is *G_12_* = 5.5 GPa. The CFRP face sheet’s density is $\rho_{c}=1.5\cdot 10^{3}{\;\mathrm{kg}/m}^{3}$. The CFRP lamina’s thickness is t* = 0.25 mm, the number of layers in the laminate is *n* = 8, the thickness of the laminate is *t_c_* (*t_c_* = nt*). The Poisson’s ratio is *ν_12_* = *ν_f_V_f_* + *ν_m_V_m_* = 0.15 [41], where *ν_f_* and *ν_m_* mean Poisson’s ratio relating to the fiber and the epoxy matrix components.

Furthermore, *V_f_* and *V_m_* mean the volume fractions relating to the fibers and the epoxy matrix. The density of the *Al* square hollow section (30 mm × 30 mm × 2 mm) stiffener is $\rho_{Al}=2.7\cdot 10^{3}{\;\mathrm{kg}/m}^{3}$.

The reduced elasticity modulus and the flexural modulus of *the CFRP* face sheet were defined by the Classical Lamination Theory (CLT). It resulted that: $E_{xred}=3.849\cdot 10^{4}\;\mathrm{MPa}$; $E_{yred}=3.222\cdot 10^{4}\;\mathrm{MPa}$; $E_{xred}^{f}=4.171\cdot 10^{4}\;\mathrm{MPa}$; $E_{yred}^{f}=3.401\cdot 10^{4}\;\mathrm{MPa}$.

#### 2.1.2. CFRP Face Sheets—Measured Elasticity Modulus and Flexural Modulus

##### Determination of Elasticity Modulus Using Tensile Test

**Figure 3:**
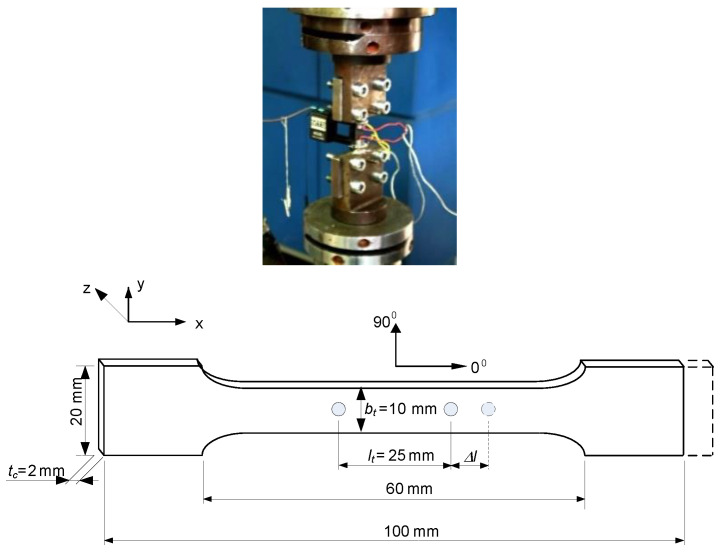
Geometry of the test specimens and the applied extensometer.

**Figure 4:**
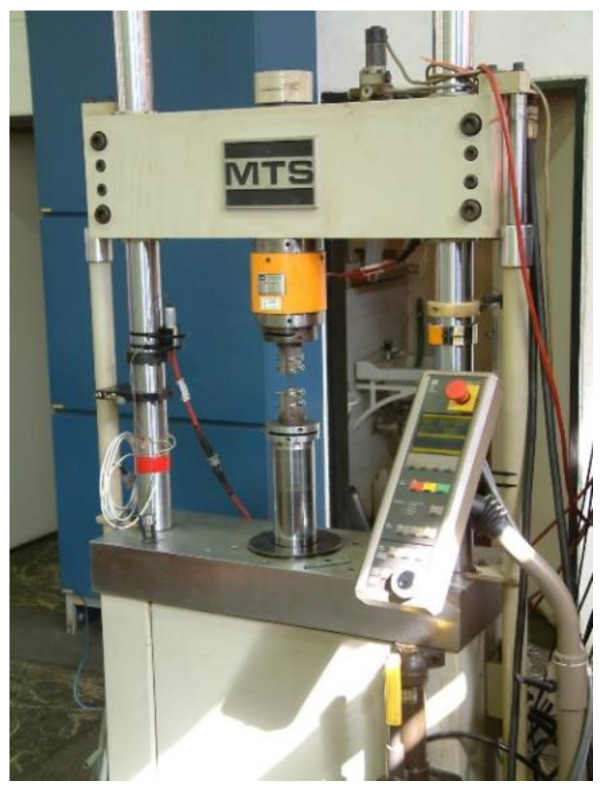
MTS tensile testing machine.

The Tensile tests of the CFRP test specimens (manufactured according to MSZ ISO 527:1993 standard) (Figure 3) were performed by the MTS tensile testing machine which capacity is 250 kN and the accuracy is 0.4–0.5% (Figure 4).

**Figure 5:**
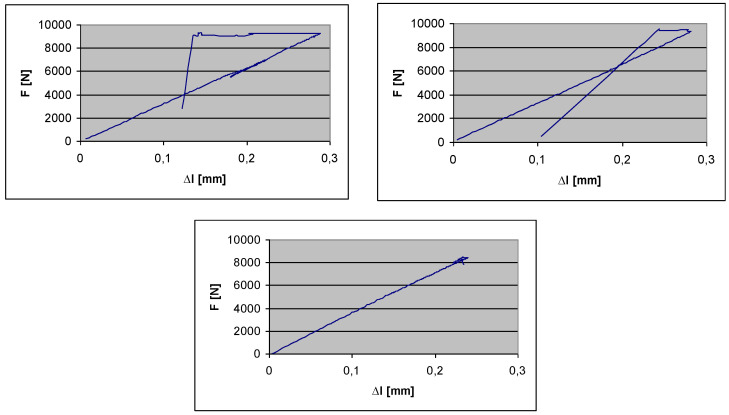
Tensile tests’ diagrams.

The maximal tensile force (*F_max_*) can be defined by the Tensile tests. Based on the test results the values of the tensile forces are near the same (9137 N) in the case of the 5 test specimens. The relevant parts of the Tensile tests’ diagrams are those parts that show where the failures of the CFRP test specimens have occurred (the highest points of the diagrams). The further parts of the diagrams show the downfalls of the extensometer after the failures of the specimens. But these parts are not relevant from the aspect of the determination of the tensile strength (*σ*_max_) (Figure 5).

● The results of the tensile tests can be seen in Figure 5.

The tensile strength (*σ*_max_) and the reduced modulus of elasticity (*E_xred_*) of the CFRP laminate can be defined by the following equations:

(1) $$\sigma_{\max}=\frac{F_{\max}}{A}=\frac{F_{max}}{b_{t}t_{c}}$$

(2) $$E_{xred}=\frac{F_{\max}l_{t}}{b_{t}t_{c}\Delta l}$$

The result of the measurements is summarized in Table 1.

##### Determination of Flexural Modulus Using Three-Point Bending Test

**Figure 6:**
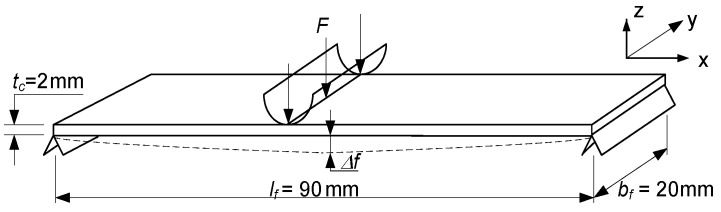
Geometry of the bending test specimens.

**Figure 7:**
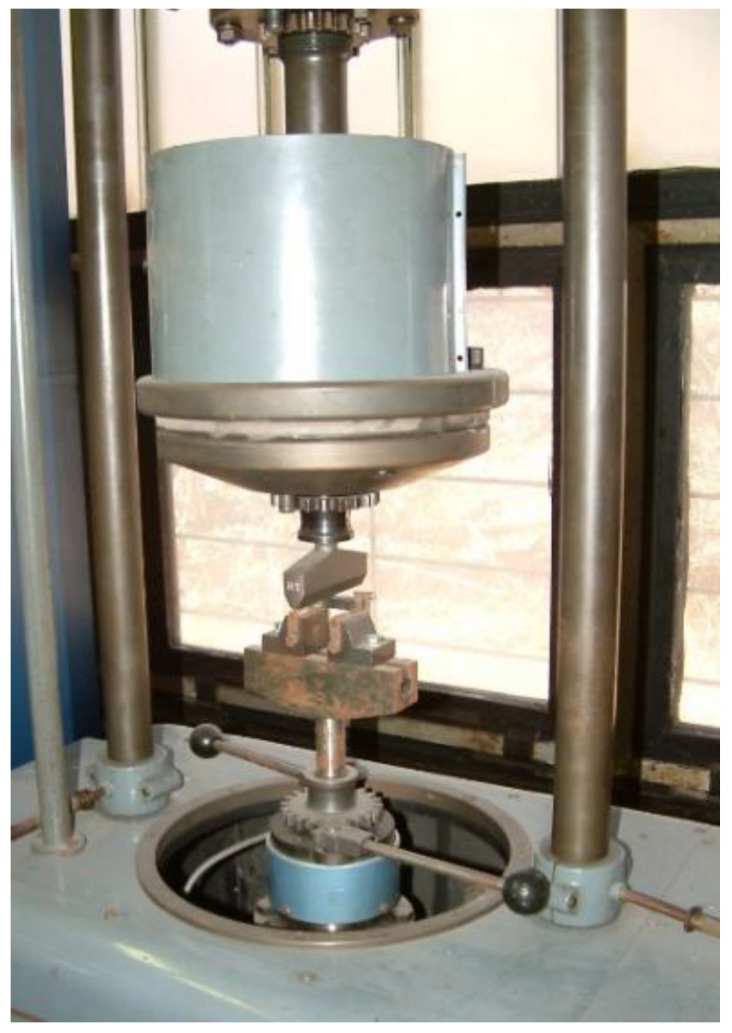
Three-point bending test machine.

The geometry of the bending test specimens (manufactured according to MSZ 892-78 standard) and the three-point bending test machine are shown in Figure 6 and Figure 7.

**Figure 8:**
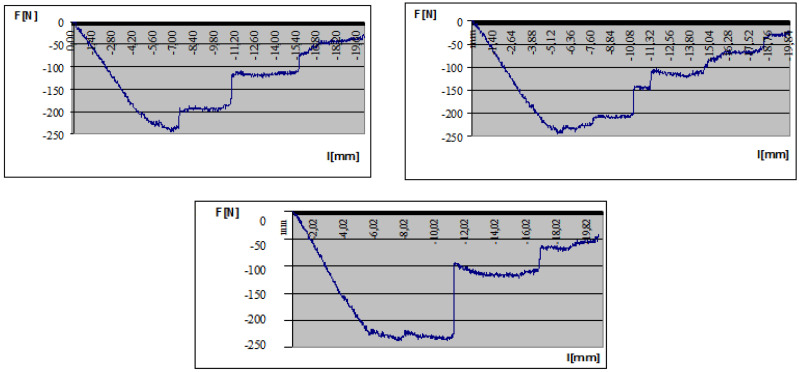
Bending tests’ diagrams.

● The results of the three-point bending tests can be seen in Figure 8.

The maximal flexural force (*F^f^*_max_) can be defined by the bending tests. Based on the test results the values of the flexural forces are near the same in the case of the 5 test specimens. The parts of the bending tests’ diagrams show the failures’ phases, when the failures occur in the individual layers of the laminates (Figure 8).

The flexural strength (σ*^f^*_max_) and the flexural modulus of the CFRP laminate (*E^f^_xred_*) can be defined by the following equations:

(3) $$\sigma_{\max}^{f}=\frac{3F_{\max}^{f}l_{f}}{2b_{f}t_{c}{}^{2}}$$

(4) $$E_{xred}^{f}=\frac{F_{\max}^{f}l_{f}^{3}}{4b_{f}t_{c}{}^{3}\cdot \Delta f}$$

The result of the measurements is summarized in Table 1.

#### 2.1.3. Comparison of the Calculated and Measured Data

The elasticity modulus and the flexural modulus of the CFRP laminate defined by the CLT theory were compared to the experimental results (Table 1).

Comparison of the results shows good agreements between the results of the calculated values and the experimental measurements of the different elastic and flexural modulus of the investigated laminated face sheets. This agreement confirms the correctness of the applied calculation methods.

### 2.2. Aluminum Square Hollow Section Stiffeners as Structural Element

**Figure 9:**
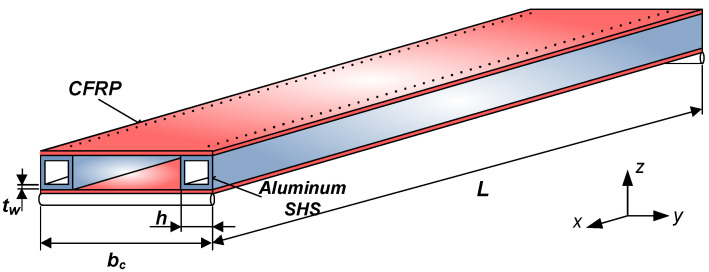
Aluminum SHS stiffeners.

The geometry of the aluminum square hollow section stiffeners (SHS) applied in the investigated structure is the following: *L* = 1200 mm, *h* = 30 mm, *t_w_* = 2 mm. The material of the Al profile is AlMgSi05, the density is *ρ_Al_* = 2.7 × 10^−6^ kg/mm^3^ (Figure 9).

### 2.3. Rivets for Joining the CFRP Face Sheets and the Al Stiffeners—Shear Test of Rivets

**Figure 10:**
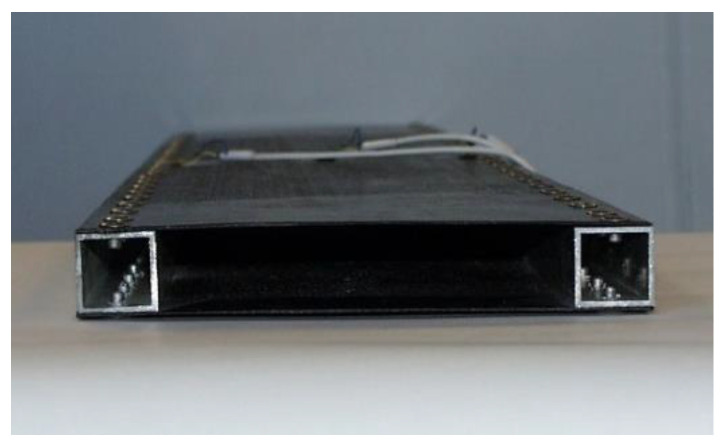
Rivets.

The connection between the face sheets and the stiffeners is provided by riveting (Figure 10).

**Figure 11:**
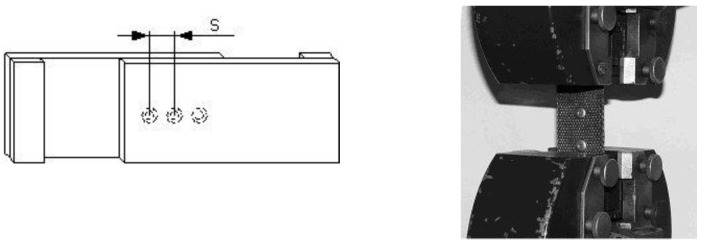
Rivets’ shear specimen.

Shear tests of the rivets were carried out to calculate the optimal number of rivets (Figure 11). The geometry of a rivet is Ø4 mm × 10 mm. The required number of rivets can be defined from the shear strength of one rivet. A shear test was completed for more rivets (1, 2 and 3). The results of the tests showed that the shear strength is linearly increasing as a function of the rivets’ number.

The adequate distance of the rivets can be defined by the following formula:

(5) $$s=\frac{F_{S}}{h\cdot \tau \cdot \gamma_{R}}=30.93\;\mathrm{mm},$$

where: *F_S_*—shear force; *h*—flange width of the Al stiffener; *τ*—shear strength of one rivet (measured value: *τ =* 1.45 MPa); $\gamma_{R}$—safety factor (=1.5).

Based on the calculation the minimal required distance between the rivets is 31 mm. The result of the calculation defines the required number of rivets that has to be applied in the case of the real manufactured sandwich-like structure.

## 3. Results—The Elaborated Calculation Methods for the Investigated Sandwich-like Structure and Validation by Experimental Measurements and Finite Element Analysis

### 3.1. Calculation Methods for the Investigated Sandwich-like Structure

#### 3.1.1. Calculation of the Middle Deflection of the Sandwich-like Structure

The total middle deflection of the investigated simple supported sandwich-like structure (Figure 1.) is the sum of the deflection (*w*) calculated by Betti’s Theorem and the deflection caused by the relative movement (Δ*w*) between the structural elements (between the face sheet and the stiffener). So the total deflection can be calculated as follows:

(6) $$w_{total}=w+\Delta w,$$

(7) $$w=\frac{ML^{2}}{12(E_{xred}I_{CFRP}+E_{Al}I_{Al})},$$

where: *M*—maximal bending moment; *E_xred_*—reduced elasticity modulus of the face sheet in *x* (longitudinal) direction; *E_Al_*—elasticity modulus of the stiffener; *I_CFRP_*—inertia moment of the CFRP face sheet; *I_Al_*—inertia moment of the Al stiffener.

The bending stiffness for the CFRP face sheet can be calculated by the following equation:

(8) $$E_{xred}I_{CFRP}=E_{xred}\left[2b_{c}t_{c}{\left(\frac{h+t_{c}}{2}\right)}^{2}\right].$$

The bending stiffness for the Al stiffener can be calculated by the following equation:

(9) $$E_{Al}I_{Al}=E_{Al}2\left[\frac{h^{3}+t_{w}}{12}+h\cdot t_{w}{\left(\frac{h-t_{w}}{2}\right)}^{2}+\frac{\left(h-\frac{t_{w}}{2}\right)t_{w}{}^{3}}{6}\right],$$

where: *b_c_*—face sheets’ width; *t_c_*—laminates’ thickness; *h*—height of the Al square stiffener; *t_w_*—wall thickness of the Al stiffener.

The measured stress data can be used to determine the relative movement’ effect between the structural elements, and can be defined as a ratio of the differences in stresses in the center of the stiffener and the face sheet. The difference of stresses ($\Delta \sigma =\left|\sigma_{Al}-\sigma_{c}\right|$) has an effect on the equivalent applied moment (Δ*M*). The relative movement caused by the sliding can be defined by the following equation:

(10) $$\Delta w=\frac{\Delta M\cdot L^{2}}{12(E_{xred}I_{CFRP}+E_{Al}I_{Al})},$$

where the difference in stress Δ*σ* results in the difference in moment Δ*M*:

(11) $$\Delta M=\Delta F(h_{Al}+\frac{nt^{*}}{2}),$$

(12) $$\Delta F=\Delta \sigma \left[nt^{*}b_{c}\right].$$

Concluding, the calculated total middle deflection of the investigated simple supported sandwich-like structure in case of a load of 500 N—based on the following equation—is 2.56 mm.

(13) $$w_{total}=w+\Delta w=1.643\;\mathrm{mm}+0.917\;\mathrm{mm}=2.56\;\mathrm{mm}.$$

#### 3.1.2. Calculation of Stresses Occurred in the Structural Components of the Analyzed Sandwich-like Structure

The applied load is distributed on the stiffeners and the face sheets. It has to be taken into consideration during the calculations of the moments and stresses that occurred in the structural elements.

This ratio can be defined by the ratio of the bending stiffness of the structural elements (*B_i_*). It can be calculated by the following formula:

(14) $$X_{i}\left[\%\right]=\frac{B_{i}}{B_{total}},$$

where: *B_i_*—bending stiffness of the Al stiffeners or bending stiffness of the CFRP face sheets; and $B_{total}=E_{Al}n_{s}I_{Al}+E_{xred}I_{CFRP}$.

The ratios of the bending stiffness of the structural elements can be calculated by the following equations:

(15) $$X_{C}=\frac{E_{xred}I_{CFRP}}{E_{Al}n_{s}I_{Al}+E_{xred}I_{CFRP}},$$

(16) $$X_{Al}=\frac{E_{Al}nI_{Al}}{E_{Al}n_{s}I_{Al}+E_{xred}I_{CFRP}},$$

where: *X_C_*—ratio of the bending stiffness relating to the CFRP face sheet; *X_Al_*—ratio of the bending stiffness relating to the *Al* stiffener; *n_s_*—number of *Al* stiffeners (=2).

Stresses occurred in the CFRP face sheet (*σ_c_*) and the *Al* stiffener (*σ_Al_*):

(17) $$\sigma_{c}=\frac{X_{C}M}{I_{CFRP}}\cdot \frac{h+t_{w}}{2},$$

(18) $$\sigma_{Al}=\frac{X_{Al}M}{n_{s}I_{Al}}\cdot \frac{h}{2}.$$

The calculated stresses in the structural elements in case of a load of 500 N are the following: $\sigma_{c}=8.434\;\mathrm{MPa}$; $\sigma_{Al}=11.503\;\mathrm{MPa}$.

### 3.2. Experimental Tests Relating to the Investigated Sandwich-like Structure

Experimental tests were performed relating to the investigated new sandwich-like structure’s middle deflection and relating to the stresses that occurred in the structural elements of the structure (CFRP face sheets and Al stiffeners).

#### 3.2.1. Experimental Tests Relating to the Sandwich-like Structure’s Deflection

**Figure 12:**
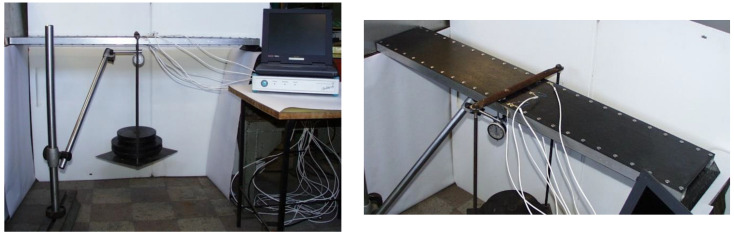
Deflection measurement.

The sandwich-like structure’s middle deflection was measured applying a displacement meter in the Al SHS stiffeners’ center lines and in the face sheet’s center point (Figure 12) in case of different loading conditions. Table 2 shows the measurements’ results.

The structure and its elements’ initial imperfections result in a difference between the deflection data relating to the two Al profiles.

#### 3.2.2. Experimental Tests Relating to the Stresses Occurred in the Structural Elements of the Sandwich-like Structure

**Figure 13:**
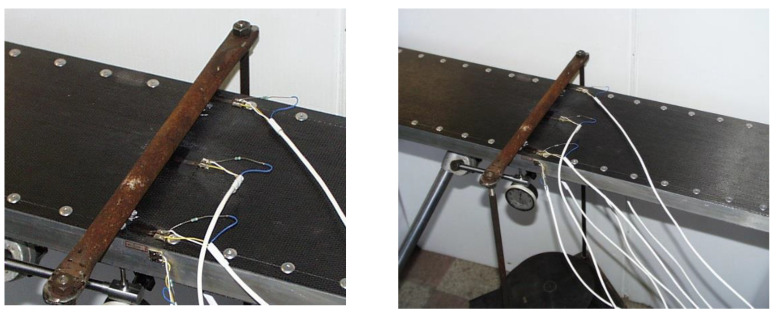
Stress measurement, strain gauges.

The stresses that occurred in the stiffeners and in the face sheets are measured applying strain gauges (accuracy is ±1%) at the investigated structure’s 7 points using 7 strain gauges (7 channels, Table 3) (Figure 13). These 7 points are located in the stiffener’s midpoint (1); in the midpoint of the face sheets’ riveting line (2, 4); in the face sheets’ midpoints (3, 6) and near to the face sheets’ riveting line (5, 7) (in that line in which the longitudinal fibers are continuous, not cut).

### 3.3. Finite Element Analysis of the Examined Sandwich-like Structure

The I-deas software was used to analyze the mechanical behavior of the investigated structure. The aim of the Finite Element (FE) analysis was the verification of the correctness of the elaborated calculation methods.

The I-deas FE software is suitable for 2 and 3 dimensional modeling, provides fast and reliable calculations and different evaluations of the gained results.

The structural elements of the investigated sandwich-like structure were defined by shell elements. The material properties were defined as an isotropic material, the laminated CFRP face sheets were defined as an orthotropic material. Eight nodes parabolic quadratic elements were applied during the FE grid generation both of Al and CFRP components. The joining of the CFRP face sheets and Al stiffeners were achieved in the same locations and modes (riveting) as in the case of the real experimental setup. In the riveting point translations in directions *x, y* and *z* are active, the rotations in directions *x, y* and *z* are inactive. Boundary conditions in the first edge (*T_x,y,z_* = 0, *R_x,y,z_* = active), in the other edge (*T_x,y_* = 0, *T_z_* = active, *R_x,y,z_* = active).

**Figure 14:**
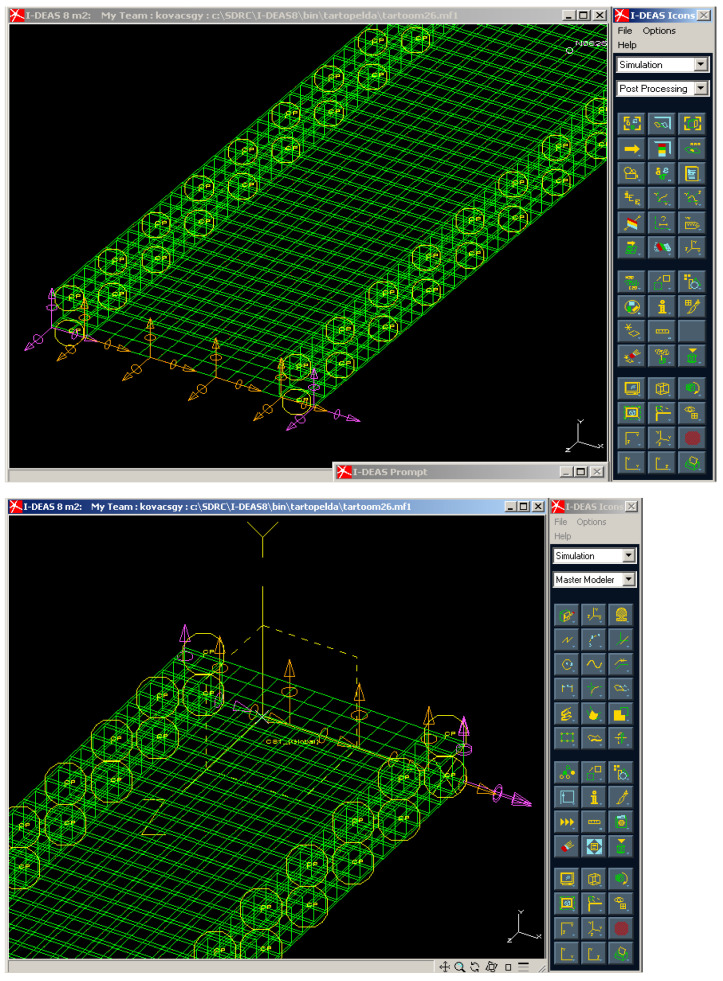
FE grid, joining of structural components, boundary conditions in the ends.

Figure 14 shows the FE grid of the examined sandwich-like structure, the joining (riveting) of structural components (laminated face sheets and stiffeners) and the boundary conditions.

Graphical and numerical evaluation of mechanical behavior of the structure in case of different loading conditions can be completed.

In the case of the examined conditions (in case of the given loading case, loading and boundary conditions and geometry) the FE calculation includes more than 1400 unknown. The program applied an iterative solution during the calculation.

**Figure 15:**
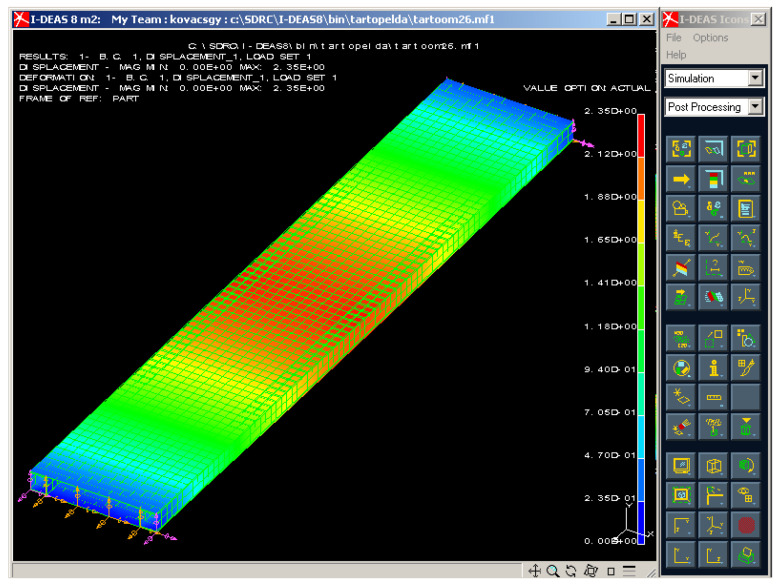
Deflection of the investigated sandwich-like structure.

Figure 15 shows the print screen of 3D graphical evaluation of the deflection of the investigated sandwich-like structure in case of the test conditions. 

Different colors depict the different values of deflections that occurred in different parts of the structure. The maximal deflection of the structure occurred in the middle line of the span where the applied load acts. The scaling of the colors provides an accurate estimation of the deflection as can be seen in Figure 15. The numerical result of the FE analysis relating to the middle deflection can be seen in Table 4.

**Figure 16:**
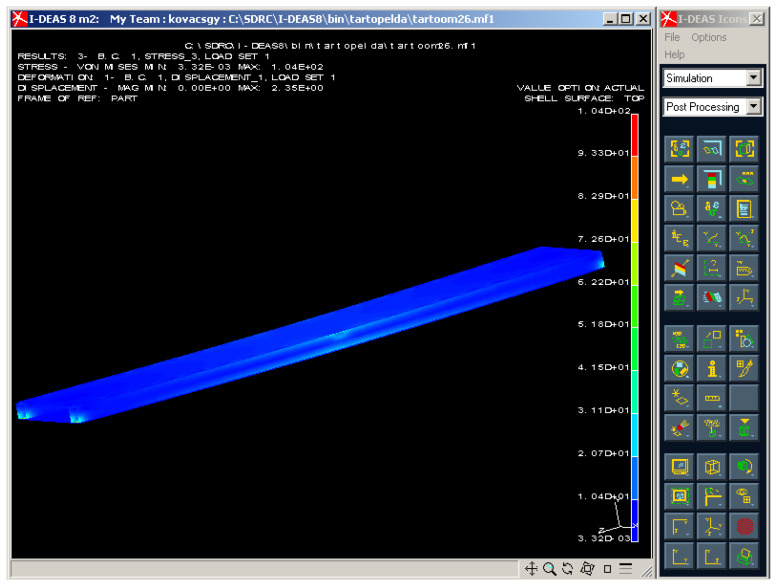
Stress distribution in the investigated sandwich-like structure.

The FE analysis was also completed for stress distribution of the structure (Figure 16). High level of stress occurred in the middle line and in the supporting points of the ends. The numerical results of the FE analysis relating to the stress in the CFRP face sheet and to the stress in the Al stiffener can be seen in Table 5.

### 3.4. Comparison of Measured, Calculated and FEM Data Relating to the Investigated Sandwich-like Structure

The gained FE results are suitable for verification of calculated and measured data relating to deflection (Table 4).

The small difference between the analytically calculated, experimentally measured and Finite Element deflections validates the analytical approximation and the elaborated calculation methods.

The calculated, measured and FE results relating to stress distribution were also compared (Table 5).

It can be summarized that there is also a small difference between the three data.

The conclusion of comparison and the differences of data confirm, that the elaborated calculation methods and the applied FE model are valid and relevant. It provides the possibility of application of the above-mentioned methods for more complex structures, e.g., multi-cellular sandwich-like structures.

## 4. Structural Optimization of the Investigated Sandwich-like Construction

The other main purpose of the study was to elaborate on the newly developed sandwich-like construction’s optimization method. During the research, the cost and the mass objective functions; furthermore, seven design constraints were elaborated and applied. The design variables were the stiffeners’ height (*h*) and the stiffeners’ wall thickness (*t_w_*) in case of the examined 4 different combinations of the CFRP layers in the laminate ([0°, +45°, −45°, 0°]_s_; [0°, 90°]_s_; [0°, 90°, 0°]; [0°, 0°]).

### 4.1. Cost Objective Function

The total cost of the construction is the sum of the material costs of the structural elements (CFRP face sheets, Al stiffeners and rivets); the heat treatment costs of the CFRP face sheets and the manufacturing costs. The cost objective function can be formulated:

(19) $$\begin{array}{c} C=C_{CFRP}+C_{Al}+C_{Rivet}+C_{heat\;treatment}+C_{manufacturing,}\\ C(\mathrm{USD})=C_{CFRP}+c_{Al}\left[n_{s}(\rho_{Al}4ht_{w}L)\right]+n_{R}c_{R}+c_{ht}+c_{f}\left[n14_{\min}+n26_{\min}+110_{\min}\right]. \end{array}$$

The highest cost is the material costs of the CFRP laminates. In the case of the investigated laminate, the layer’s specific material cost is *C_CFRP_* = 26 USD/m^2^. The heat treatment costs of CFRP face sheets is depending on the dimension of the face sheets and the characteristics of the Epoxy ES-67 resin. In the case of the investigated laminate, the heat treatment’s total cost is USD 4. The specific material cost of the Al stiffener is *c_Al_* = 4.94 USD/kg. The rivet’s specific material cost is *c_R_* = 0.01 USD/pcs. The *n_s_* is the number of stiffeners; the *n_R_* is the number of rivets. The specific manufacturing cost is *c_f_* = 0.6 USD/min. The construction’s total manufacturing cost is the sum of the CFRP face sheets’ manufacturing costs, the cutting costs of the *Al* stiffeners and the total cost of the assembly of the structural elements.

The manufacturing cost can be defined as the function of the lead times of the manufacturing activity (in minutes) joining to the manufacturing of the CFRP face sheets. This manufacturing time is including the procedure of the following manufacturing processes: press form preparation, cutting of the laminas, sequencing of the laminas and finishing processes. The other component of the total manufacturing time is the final assembly of the structural elements including the face sheets’ and stiffeners’ drilling; furthermore, the time consumption of the riveting.

### 4.2. Mass Objective Function

The most important design aim—in the case of the application of FRP composite materials—is the reduction of the total weight of the construction.

The analyzed sandwich-like structure’s total weight is the sum of the structural elements’ weights (face sheets, stiffeners and rivets) (Figure 1).

(20) $$m=2\rho_{c}\left[b_{c}L(nt^{*})\right]+n_{s}\rho_{Al}\left[L4(ht_{w}-t_{w}^{2})\right]+n_{R}\rho_{R}.$$

### 4.3. Design Constraints

During the structural optimization the following seven design constraints were considered:

**1.** Total deflection of the structure, which can be calculated by the following equation:

(21) $$w_{\max}=\frac{ML^{2}}{12(E_{xred}I_{CFRP}+E_{AL}I_{AL})}+\frac{\Delta M\cdot L^{2}}{12(E_{xred}I_{CFRP}+E_{AL}n_{s}I_{AL})}\leq \frac{L}{200}.$$

**2.** Buckling of the CFRP face sheet, which can be calculated by the following equation:

(22) $$\left(\frac{b_{c}}{nt^{*}}\right)\leq \sqrt{\frac{\pi^{2}}{6\sigma_{\max}\left(1-\nu_{xy}^{f}\nu_{yx}^{f}\right)}\left[\sqrt{E_{x}^{f}E_{y}^{f}}+E_{x}^{f}\nu_{xy}^{f}+2G_{xy}^{f}\left(1-\nu_{xy}^{f}\nu_{yx}^{f}\right)\right]}.$$

where: $\sigma_{\max}$—maximal stress in the CFRP laminate; $E_{x}^{f},E_{y}^{f},G_{xy}^{f},\nu_{xy}^{f},\nu_{yx}^{f}$—flexural parameters of the laminate [41].

**3.** Web buckling in the Aluminum stiffeners, which can be calculated by the following equation:

(23) $$\frac{h}{t_{w}}\leq 42\sqrt{\frac{235E_{Al}}{240E_{Steel}}},$$

where: *E_Steel_*—Steel’s elasticity modulus [42]

**4.** Stress in CFRP face sheets, which can be calculated by the following equation:

(24) $$\sqrt{\left(\sigma_{L}^{2}+\sigma_{T}^{2}+\sigma_{L}^{}\sigma_{T}^{}\right)}\leq \sigma_{Call},$$

where: *σ_L_*—stress caused by longitudinal bending; *σ_T_*—stress caused by transverse bending; *σ_Call_*—CFRP face sheet’s allowable tensile strength; $\sigma_{Call}=\sigma_{Tc}/\gamma_{c}$, $\sigma_{Tc}$—CFRP face sheet’s tensile strength; $\gamma_{c}$—safety factor (=2).

Stress caused by bending (in longitudinal direction) can be calculated by the following equation:

(25) $$\sigma_{L}=\frac{X_{C}M}{I_{CFRP}}\cdot \frac{h+nt^{*}}{2},$$

where: $X_{C}M$—moment occurred on the CFRP face sheet; $X_{C}=\frac{E_{xred}I_{CFRP}}{E_{Al}n_{s}I_{Al}+E_{xred}I_{CFRP}}$.

Stress caused by transversal bending can be calculated by the following equation:

(26) $$\sigma_{T}=\frac{M}{\frac{{\left(nt^{*}\right)}^{2}}{6}}.$$

**5.** Stress in stiffeners, which can be calculated by the following equation:

(27) $$\frac{X_{Al}M}{n_{s}I_{Al}}\cdot \frac{h}{2}\leq \sigma_{Alall},$$

where: $X_{Al}M$—moment occurred on stiffener; $X_{Al}=\frac{E_{Al}n_{s}I_{Al}}{E_{Al}n_{s}I_{Al}+E_{xred}I_{CFRP}}$; $\sigma_{Alall}=\frac{f_{y}}{\gamma_{Al}}$—allowable stress; $f_{y}$—Aluminum’s yield stress; $\gamma_{Al}$—safety factor (=1.5).

**6.** Structure’s eigenfrequency, which can be calculated by the following equation:

(28) $$f_{1}=\frac{\pi }{2L^{2}}\sqrt{\frac{10^{3}(E_{Al}I_{Al}+E_{xred}I_{CFRP})}{m}}\geq f_{0},$$

where: *m*—construction’s mass/unit length [kg/m]; *f_0_*—allowable eigenfrequency (50 Hz).

**7.** Size constraints relating to design variables are the following:

(29) $$\begin{array}{c} 10\leq h\leq 100\;[\mathrm{mm}],\\ 2\leq t_{w}\leq 6\;[\mathrm{mm}]. \end{array}$$

### 4.4. Flexible Tolerance Optimization Algorithm

The optimization of the newly elaborated sandwich-like structure was achieved by the Flexible Tolerance Optimization method, which is a constrained random search method developed by Himmelblau [43,44]. The Flexible Tolerance Optimization algorithm improves the value of the objective function based on the information of feasible points and near-feasible points.

(30) $$\begin{array}{c} \mathrm{Minimize:}\;\mathrm{f(x)},\\ \mathrm{Subject}\;\mathrm{to}:\;\Phi^{\left(k\right)}-T(x)\geq 0 \end{array}$$

where: $\Phi^{\left(k\right)}$—flexible tolerance criteria for viability at stage *k*; *T(x)*—positive function for design constraints.

### 4.5. Results of the Structural Optimization for Different Face Sheets’ Layer Sequences

**Figure 17:**
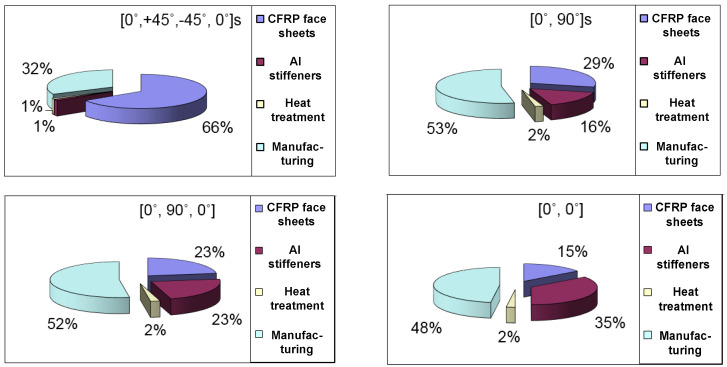
Cost components of the optimal sandwich-like structures in case of 4 different face sheets’ layer combinations.

**Figure 18:**
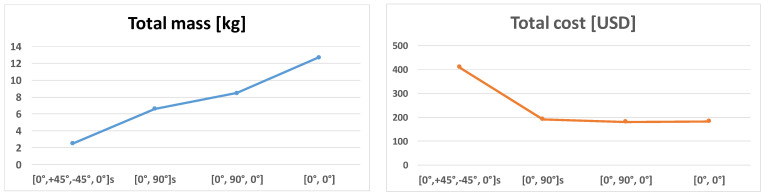
Total mass and total cost of the optimal sandwich-like structures in case of different face sheets’ layer combinations.

The optimization was carried out for four different layer sequences of the face sheets. These four different face sheets are the following: the manufactured [0°, +45°, −45°, 0°]_s_; [0°, 90°]_s_; [0°, 90°, 0°] and [0°, 0°] layer sequences. The design variables were the stiffeners’ height (*h*) and the stiffeners’ wall thickness (*t_w_*) in the case of the examined 4 different combinations of the CFRP layers. The mass and the cost structural optimization were carried out for the before mentioned 4 different face sheets (Figure 17 and Figure 18).

The optimization results are tabulated in Table 6 which are obtained by the application of the Flexible Tolerance optimization method. During the optimization the mass and the cost objective functions (Equations (19) and (20)); furthermore, the before mentioned 7 design constraints (Equations (21)–(29)) were considered. The same optimal heights and wall thicknesses are obtained for the optimal Al stiffeners in the case of both objective functions for each of the different face sheets’ layer sequences. It means that the obtained optimal constructions in case of the different layer sequences represent the most light-weighted and the most cost-effective, economic structures.

The most often used fiber orientations are the 0°, 45° and 90° in industrial applications. The results of the preliminary calculations and the optimization (Table 6) showed that the [0°, +45°, −45°, 0°]_s_ layer sequence provides the most light-weighted sandwich-like structure. The primary aim of the optimization was weight saving. It was the reason that the above-mentioned layer combination was manufactured and investigated during the measurements.

The results of the optimization show that the most light-weighted sandwich-like structure (2.498 kg) can be constructed by the application of a CFRP laminate which has 8 layers; furthermore, the layer sequence is the following [0°, +45°, −45°, 0°]_s_. The optimal geometry of the *Al* stiffeners is 25 mm × 25 mm × 1.5 mm (Table 6, Figure 18).

The optimization results also show that the most cost-effective sandwich-like structure (USD 180.958) can be constructed by the application of a *CFRP* laminate which has 3 layers; furthermore, the layer sequence is the following [0°, 90°, 0°]. The optimal geometry of the *Al* stiffeners is 80 mm × 80 mm × 4 mm (Table 6, Figure 18).

## 5. Conclusions

● The main findings and the results of the research are the following:

**(1)** A new light-weight sandwich-like structure was constructed, which consists of CFRP face sheets and Al stiffeners (Figure 1). The face sheets are riveted to the stiffeners. This new structure was manufactured in order to take experimental measurements (Figure 12).

The new sandwich-like structure is the combination of different materials (CFRP and Al) and structural elements (elements of sandwich structures and cellular plates); because the face sheets made of CFRP composite (as in the case of sandwich structures); furthermore, the stiffeners made of metal (as in case of cellular plates).

**(2)** Before the elaboration of the calculation methods for the newly constructed sandwich-like structure, preliminary calculations and experimental measurements (Figure 5, Figure 8 and Figure 11) had to be carried out relating to the structural elements (face sheets, stiffeners and rivets).

Then calculation methods were elaborated for the middle deflection of the new sandwich-like structure; at the same time for the stresses that occurred in the structural elements.

After it experimental measurements (Table 2 and Table 3) and Finite Element analysis (Figure 15 and Figure 16) were achieved. The results of the calculations, measurements and Finite Element analysis were near the same which confirmed the correctness of the elaborated calculation methods (Table 4 and Table 5).

**(3)** Mass and cost optimization methods were elaborated for the investigated construction. During the optimization the Flexible Tolerance optimization method was applied considering the following seven design constraints: total deflection; buckling of face sheet; web buckling in stiffeners; stress in CFRP face sheets; stress in Al tubes; construction’s eigenfrequency; constraints relating to the design variables. The results of the mass and the cost optimization were summarized in Table 6.

● The main added values of the research are the following:

**(1)** The novelty of the study is the elaboration of the calculation methods on the one hand for the middle deflection of the investigated new sandwich-like structure; on the other hand, for the stresses that occurred in the structural elements. The elaborated calculation methods were validated by experimental measurements and Finite Element analysis.

**(2)** The other main contribution of the research is the elaboration of the mass and cost optimization method considering seven design constraints applying the Flexible Tolerance optimization method. But the primary aim of the optimization was to construct the most light-weighted structure.

The newly constructed light-weight sandwich-like structure can be widely used in many industrial applications where the primary design aim is weight saving, e.g., elements of vehicles (ship floors, airplane base plate, etc.); transport containers; building constructions (building floors, bridge decks, etc.).

In future research—based on the elaborated calculation methods—more complex structures can be investigated and optimized for other engineering applications. Furthermore, additional design constraints and other structural elements can be used during the optimization.

## Figures and Tables

**Table 1:** Comparison of the calculated and the measured data.

	Calculated	Measured	Difference (%)
**Elastic modulus**	38,490 MPa	41,650 MPa	8.2
**Flexural modulus**	41,710 MPa	42,478 MPa	1.84

**Table 2:** Results of the deflection measurement.

Loading Conditions (N)					
Middle Deflection (mm)	300 N	400 N	500 N	600 N	700 N
1st Al stiffener	1.61	1.96	2.3	2.65	3.0
Face sheet midpoint	1.78	2.19	2.59	2.98	3.41
2nd Al stiffener	1.8	2.2	2.65	3.0	3.43
Average in Al stiffener	1.705 ± 0.095	2.08 ± 0.12	2.475 ± 0.175	2.825 ± 0.175	3.215 ± 0.215

**Table 3:** Results of the stress measurement.

Loading Conditions (N)				
Stresses (MPa)	300 N	400 N	500 N	600 N
Channel **1**	−7.172	−9.73	**−12.208**	−14.97
Channel 2	−6.647	−8.568	−10.291	−11.302
Channel 3	−4.915	−6.346	−7.788	−9.242
Channel 4	−5.097	−6.667	−8.381	−9.358
Channel **5**	5.072	7.015	**8.859**	10.503
Channel 6	4.624	6.111	7.622	9.024
Channel **7**	4.796	6.497	**8.259**	9.993

**Table 4:** Comparison of middle deflection data of the analyzed structure.

	Middle Deflection (mm)	Ratio	Difference (%)
**Measured**	2.475	100%	0
**Calculated**	2.56	103.43%	+3.43
**FEM**	2.35	94.95%	−5.05

**Table 5:** Comparison of data relating to the occurred stresses in the investigated structure in case of 500 N (load).

	Location	Stress (MPa)	Ratio	Difference (%)
**Measured**	Stress in the CFRP face sheet	8.559 MPa	100%	0
	Stress in the Al stiffener	12.208 MPa	100%	0
**Calculated**	Stress in the CFRP face sheet	8.434 MPa	98.54%	−1.46%
	Stress in the Al stiffener	11.503 MPa	94.22%	−5.78%
**FEM**	Stress in the CFRP face sheet	9.305 MPa	108.72%	−8.72%
	Stress in the Al stiffener	11.12 MPa	91.09%	−8.9%

**Table 6:** Results of the structural optimization.

Layer Sequence of the Face Sheet	Optimal Al Geometries		Total Mass (kg)	Total Cost (USD)	Cost Components	
	*h* (mm)	*t_w_* (mm)				
[0°, +45°, −45°, 0°]_s_	25	1.5	**2.498**	410.002	270.4	CFRP face sheets
					4.802	Al stiffeners
					4	Heat treatment
					130.8	Manufacturing
[0°, 90°]_s_	60	4	6.598	191.633	54.912	CFRP face sheets
					30.721	Al stiffeners
					4	Heat treatment
					102	Manufacturing
[0°, 90°, 0°]	80	4	8.474	**180.958**	41.184	CFRP face sheets
					40.974	Al stiffeners
					4	Heat treatment
					94.8	Manufacturing
[0°, 0°]	100	5	12.708	183.078	27.456	CFRP face sheets
					64.022	Al stiffeners
					4	Heat treatment
					87.6	Manufacturing

## Data Availability

Not applicable.
